# Make it double: identification and characterization of a Tandem-Hirudin from the Asian medicinal leech *Hirudinaria manillensis*

**DOI:** 10.1007/s00436-022-07634-0

**Published:** 2022-08-25

**Authors:** Phil Lukas, Georgij Melikian, Jan-Peter Hildebrandt, Christian Müller

**Affiliations:** Animal Physiology, Zoological Institute and Museum, University of Greifswald, Felix-Hausdorff-Str. 1, D-17489 Greifswald, Germany

**Keywords:** Hirudin, Hirudin-like factors, *Hirudinaria manillensis*, Blood coagulation, Medicinal leeches, Tandem-Hirudin

## Abstract

Haematophagous leeches express a broad variety of secretory proteins in their salivary glands, among them are hirudins and hirudin-like factors. Here, we describe the identification, molecular and initial functional characterization of Tandem-Hirudin (TH), a novel salivary gland derived factor identified in the Asian medicinal leech, *Hirudinaria manillensis*. In contrast to the typical structure of hirudins, TH comprises two globular domains arranged in a tandem-like orientation and lacks the elongated C-terminal tail. Similar structures of thrombin inhibitors have so far been identified only in kissing bugs and ticks. Expression of TH was performed in both cell-based and cell-free bacterial systems. A subsequent functional characterization revealed no evidence for a thrombin-inhibitory potency of TH.

## Introduction

Haematophagous animals, no matter whether insects, ticks, leeches or even mammals, face the same fundamental challenges during their blood meals on vertebrate hosts: staying undetected, getting enough food and preventing premature haemostasis (Ware and Luck 2017). For the latter, a broad arsenal of bioactive compounds has been developed by nature including inhibitors of platelet aggregation (primary haemostasis) and inhibitors of blood coagulation (secondary haemostasis) (Ware and Luck 2017; Cherniack 2011; Klotz et al. 2014; Oka et al. 1989; Li et al. 2018; Manship et al. 2012). Putative targets of blood coagulation inhibitors are factor Xa (Stuart–Prower factor) and factor IIa (thrombin) (Koh and Kini 2010, 2009). Hirudin is probably the best known natural direct thrombin inhibitor and was originally identified in the European medicinal leech, *Hirudo medicinalis* (Markwardt 1955, 1970). However, several other thrombin inhibitors have been described in leeches (haemadin), ticks (boophilin, ornithodorin and variegin), kissing bugs (rhodniin and triabin) and mosquitoes (anophelin), respectively (Strube et al. 1993; Macedo-Ribeiro et al. 2008; Friedrich et al. 1993; Mans et al. 2002; Oka et al. 1989; Koh et al. 2007; Noeske-Jungblut et al. 1995). Hirudin and haemadin share a common structure with a short N-terminal part, one central globular domain stabilized by three disulfide bridges and an elongated C-terminal tail (Grütter et al. 1990; Richardson et al. 2000). In contrast, boophilin, ornithodorin and rhodniin show a tandem-like structure with two globular domains connected by a short linker and without an elongated tail (Macedo-Ribeiro et al. 2008; Friedrich et al. 1993; Mans et al. 2002). Strikingly, the globular domains are stabilized by three disulfide bonds as well, yet in a completely different way: Cys1-Cys2, Cys3-Cys5 and Cys4-Cys-6 in hirudin and haemadin; Cys1-Cys6, Cys2-Cys4 and Cys3-Cys5 in boophilin and ornithodorin; and Cys1-Cys5, Cys2-Cys4 and Cys3-Cys6 in rhodniin, respectively (Grütter et al. 1990; Richardson et al. 2000; Macedo-Ribeiro et al. 2008; Friedrich et al. 1993; Mans et al. 2002). A tandem-like structure has been described for leech-derived factors so far only for members of the antistasin-family like antistasin itself and ghilanten (Whitlow et al. 1999). Both are inhibitors of factor Xa (Blankenship et al. 1990; Dunwiddie et al. 1989). We have recently described the identification and functional characterization of a novel class of leech-derived compounds, the hirudin-like factors (HLFs), in members of the genera *Hirudo* and *Hirudinaria* (Lukas et al. 2019; Müller et al. 2016, 2017, 2019a). HLFs comprise structural features characteristic to hirudin (e.g. six cysteine residues within a central globular domain and a common gene structure composed of four exons and three introns), but may considerably differ in biochemical properties like molecular weight (MW) and isoelectric point (pI value) (Müller et al. 2017). Some of the HLFs show thrombin-inhibitory potencies as high as hirudin (e.g. HLF1long of *Hirudo orientalis*), whereas others have reduced inhibitory capacities (e.g. HLF5 and HLF8 of *Hirudinaria manillensis*) or a very low or even no detectable potencies at all (e.g. HLF1D of *Hirudo verbana* and HLF6 of *Hirudinaria manillensis*) (Lukas et al. 2019; Müller et al. 2019a). All these factors were identified prior to the availability of genomic or transcriptomic data by either cDNA library screening or targeted and non-targeted PCR approaches. As an unexpected outcome of such a reaction, we identified a cDNA that encodes a yet unknown putative salivary gland-derived factor of *Hirudinaria manillensis* with two hirudin-like globular domains arranged in a tandem-like orientation. We therefore named this factor Tandem-Hirudin (TH). The structure of TH resembles those of the thrombin inhibitors of ticks and kissing bugs mentioned above, and we hence derived the hypothesis that TH might be an inhibitor of thrombin as well. In the present study, we describe the structure and our attempts to express and functionally characterize TH.

## Material and methods

### Genotyping of animals and tissue preparation

The biological material used in this study (specimen of *Hirudinaria manillensis* and salivary gland preparations) was already described by Müller et al. (2017). Information on genotyping data and GenBank accession numbers can be obtained from the respective publication.

### Sequence analysis

Nucleotide and amino acid sequence alignments were generated using the CLS Sequence Viewer software package v8.0 (CLC bio, Aarhus, Denmark) with the following parameters: gap open cost, 5.0; gap extension cost, 2.0; end gap cost, free. Alignments were exported as msf-files and further processed using GeneDoc v2.7 (Nicholas and Nicholas 1997). Signal peptide sequences were predicted using the Phobius web server (Käll et al. 2007) and SignalP6.0 (Teufel et al. 2022).

### Isolation and cloning of a Tandem-Hirudin cDNA

Tissue preparation, RT-PCR and primer design were already described in detail elsewhere (Müller et al. 2017). For bacterial expression, the cDNA of TH without its putative signal peptide coding sequence was cloned into the bacterial expression vector pQE30Xa (Qiagen, Hilden, Germany). In addition, a plasmid for cell free protein synthesis of TH was generated as well (see below).

### Bacterial expression, sulfitolysis and refolding of TH

The *E. coli* strain GM1674 (Palmer and Marinus 1994) was used for bacterial expression of TH. Expression and purification of TH were performed as described by Lukas et al. (2019). However, the successful downstream processing of TH required a partial sulfitolysis of the protein prior to the factor Xa-cleavage. The sulfitolysis was performed by modified methods obtained from Lundblad (1995) and Grant (2017). Tandem-Hirudin was first dialysed overnight against 0.5 L of a sodium sulfite buffer (0.05 mol/l sodium sulfite, 0.2 mol/l cysteine-HCl, 8 mol/l urea, 0.1 mol/l Tris–HCl, pH 7.0) at room temperature in a 5 ml micro-dialysis capsule (QuixSep, Roth, Karlsruhe, Germany) and a dialysis membrane with a molecular mass cut off (MWCO) of 5,000 Da (Roth, Karlsruhe, Germany). After dialysis the capsule was exposed to air for 4 h at room temperature. For refolding, the capsule was placed in 1 L of refolding buffer (50 mmol/l Tris–HCl, 400 mmol/l sucrose, 10% (v/v) glycerol, 0.5% (v/v) Triton X-100, pH 8.0, modified after Moghadam et al. (2015) and dialysed for another 48 h with one buffer replacement after 24 h. A fresh volume of refolding buffer was degassed and enriched with 3 mmol/l glutathion under nitrogen atmosphere. The capsule was incubated for another 4 h at room temperature and constant stirring. After incubation, the nitrogen was replaced with air, and 0.3 mmol/l glutathione disulfide was added, and the capsule incubated for 20 h at 4 °C. A final dialysis was performed against reaction buffer (20 mmol/l Tris/HCl, 100 mmol/l NaCl, 2 mmol/l CaCl_2_, pH 8.0) for 24 h at 4 °C and repeated for another 24 h. Factor-Xa cleavage was performed for 48 h at room temperature (Lukas et al. 2019; Müller et al. 2017).

### Cell-free synthesis of TH in bacterial cell lysates

For the cell-free synthesis of TH in bacterial cell lysates, the NEBExpress® Cell-free *E. coli* Protein Synthesis System (New England Biolabs, Frankfurt a. M., Germany) was applied. Expression of proteins was achieved by a coupled transcription (T7 RNA polymerase supplied with the kit) and translation process. The coding sequences for TH as well as hirudin variant HV1 and HLF5 (both factors served as positive controls) were cloned into the expression vector pET-28a( +) (Merck Millipore, Darmstadt, Germany) using the unique *Nco*I restriction site at position 296 to generate an appropriate start codon for translation. As a consequence, the N-terminal amino acid sequence of hirudin variant HV1 was changed from VVYTDC to MVYTDC. The N-terminal amino acid sequence of TH was accordingly modified from VCTVC to MVYTDC. All further steps of the synthesis reactions followed the instructions and recommendations as outlined by the manufacturer. Briefly, 250 ng of plasmid DNA in a total reaction volume of 50 µl were incubated for 4 h at 37 °C in an orbital shaker (ThermoMixer C, Eppendorf, Hamburg, Germany). The empty vector pET-28a( +) and the DHFR-His control template (supplied with the kit) were used for control purposes in separate protein synthesis reactions. To support a proper disulfide bond formation the PURExpress® Disulfide Bond Enhancer (New England Biolabs, Frankfurt a. M., Germany) was added. Aliquots of all synthesis reactions were analysed by SDS-PAGE on a 20% gel.

### Coagulation assays

To verify the putative anti-coagulatory activity of TH, three blood coagulation assays were performed: the activated partial thromboplastin time test (aPTT; reference range 22.7–28.9 s), the prothrombin time test (PT; reference range 10.7–13.7 s) and the thrombin time test (TT; reference range 16.8–21.4 s) using a BFT II Analyzer (Siemens Healthcare, Erlangen, Germany). All steps followed the instructions outlined by the manufacturer. For the coagulation assays, the protein samples were either diluted with dialysis buffer to reach final concentrations of 50 µmol/l or 5 µmol/l, respectively, or used in the highest concentration possible. The desired amount of substrate was transferred directly into the cuvette immediately before the plasma was added. Dade® Ci-Trol® 1 (Siemens Healthcare, Erlangen, Germany) was used as standardised human plasma. The incubation of reaction mixtures was carried out at 37.4 °C. Measurements that lasted up to 300 s were stopped and declared as a complete inhibition of coagulation. Clot formation was additionally controlled by eye to exclude technical errors. All samples where measured immediately after cell-free synthesis. TH expressed in bacteria was kept in aliquots and thawed on ice immediately before the measurements.

### Platelet aggregation assay

The platelet aggregation assays were performed as already described in detail by Lukas et al. (2019).

For TH expressed and purified from bacteria, 10 µl of protein with a concentration of 75 µmol/l were transferred into the cuvettes, and 180 µl PRP were added, resulting in a final concentration of 3.8 µmol/l. Ten microlitre of dialysis buffer served as the control. Eptifibatide in an equimolar amount was used as a positive control for inhibition of platelet aggregation. Output parameters were calculated as described (Lukas et al. 2019).

### Trypsin activity assay

The putative effects of TH on the trypsin activity were evaluated by a chromogenic assay slightly modified after Erlanger et al. (1961). Briefly, wells on a transparent 96 well plates (Sarstedt, Nümbrecht, Germany) were prefilled with 22.4 µl of either dialysis buffer (negative control) or sample solution to give a final concentration of 0.4 or 3.8 µmol/l. Afterwards, 9.6 µl of a standard trypsin solution used for cell culture purposes (2.5 g/l, PAN-Biotech, Aidenbach, Germany) was added. Equimolar amounts of the soybean trypsin inhibitor (SBTI, AppliChem, Darmstadt, Germany) were used as a positive control. The samples were incubated for 5 min at 37 °C followed by the addition of 167 µl of substrate solution (1 mmol/l N α-benzoyl-L-arginine-4-nitroanilide hydrochloride (BAPA) in 0,01% dimethyl sulfoxide (DMSO), 50 mmol/l Tris–HCl, 20 mmol/l CaCl_2_, pH = 8.2). Every 30 s, the absorbance at a wavelength of λ= 410 nm was measured over a period of 5 min in a standard plate reader (Tecan, Crailsheim, Germany). Calculation of trypsin activity followed an adapted equation of Sørensen et al. (1999):

U/mg = (A_410_/min ∙ GV ∙ VF)/(ε ∙ d ∙ PV ∙ mg/ml).

(*A*_*410*_*/min* is the alteration of the extinction at λ= 410 nm per minute, *GV* is the whole sample volume in ml, *VF* is the dilution factor, *ε* is the molar attenuation coefficient in cm^2^/µmol, *d* is the thickness of the cuvette in cm, *PV* is the amount of trypsin solution in ml, and *mg/ml* is the concentration of trypsin solution).

## Results

### Identification and characterization of a TH encoding cDNA

**Fig. 1:**
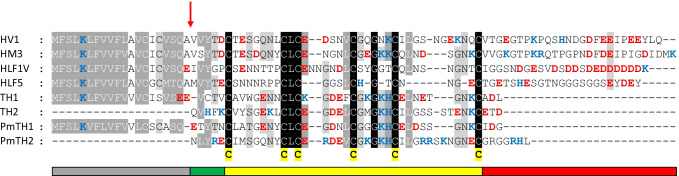
Multiple sequence alignment of hirudin-variant 1 (HV1) and hirudin-like factor HLF1V from *Hirudo medicinalis*, hirudin-variant HM1 and HLF5 from *Hirudinaria* (*Poecilobdella*) *manillensis* and domain-I (TH1) and domain-II (TH2) of Tandem-Hirudins TH and PmTH (a putative TH encoded by *Poecilobdella manillensis*). Black background indicates conserved residues; grey background indicates similar residues. Acidic amino acid residues are labelled in red, basic amino acid residues in blue. A red arrow indicates the signal peptide cleavage site. The six conserved cysteine residues giving rise to the three dimensional structure are marked in bold and labelled in yellow. Boxes indicate the structural and functional domains of hirudin HV1: signal peptide (grey), N-terminus (green), central globular domain (yellow) and elongated C-terminal tail (red). Abbreviations are used according to the IUPAC code

Isolation of cDNA of TH followed the same general protocol as already described for the identification of recently reported HLFs (Müller et al. 2016, 2017, 2019a). Extracts of RNA derived from two *Hirudinaria manillensis* individuals were analysed by reverse transcription polymerase chain reaction (RT-PCR) with a forward primer that spans a portion of the coding region of the signal peptide and an oligo dT-primer. Amplified fragments were cloned, and sequences of individual clones were determined. Sequence analyses of two clones revealed inserts with lengths of 572 bp (clone 1) or 517 bp (clone 2), respectively, including a polyA-stretch. Clone 1 contains an additional stretch of 56 nucleotides immediately before the polyA tail but is otherwise identical to clone 2. Sequence data were deposited in GenBank under Acc. No. MZ361731 and MZ361732. Both sequences contain open reading frames of 306 bp (including the stop codon) that encode a putative protein of 101 amino acid residues in length including a signal peptide sequence. The derived amino acid sequences revealed the presence of 13 cysteine residues. With exception of the first, all cysteine residues were arranged in a manner (position and spacing) typical for the central globular core domain of hirudins and HLFs (Fig. 1).

In addition, the arrangement of cysteine residues clearly pointed to a tandem-like duplication of the central globular domain in the putative new protein, and the factor was hence termed Tandem-Hirudin (TH). Detailed comparison of the central globular domains (Cys1-Cys6) of representatives of hirudins and HLFs with the two domains of TH (TH1 and TH2) revealed degrees of sequence identity/similarity between 29–65% and 38–78%, respectively (Table 1).

### Molecular properties of TH

Hirudins and HLFs of *Hirudo* spec. and *Hirudinaria manillensis* contain highly conserved signal peptide sequences of 20 amino acid residues in length. The putative signal peptide sequence of TH almost perfectly fits into that scheme (see Fig. 1); however, cleavage site predictions using several online software tools led to heterogeneous outcomes, most probably due to the presence of the two glutamic acid residues at positions 19 and 20.

Hirudins and HLFs with thrombin-inhibiting activity exhibit several characteristic molecular features including an acidic pI value of about 3.2—4.6 (Müller et al. 2019a, 2017). The low value is mainly caused by several acidic amino acid residues within the elongated C-terminal tail, but the central globular domain (Cys1-Cys6) as well has to be acidic for inhibitory activity (Müller et al. 2019a). Analyses of the TH1 and TH2 domains revealed a length of 32 or 34 amino acid residues and pI values of 5.59 and 4.96, respectively (Table 2).

**Table 1:** Comparison of the central globular domains (Cys1-Cys6) of hirudins (hirudin variants HV1 of Hirudo medicinalis and HM3 of Hirudinaria manillensis)and hirudin-like factors (HLF1V of Hirudo medicinalis and HLF5 of Hirudinaria manillensis) with the two domains of TH (TH1 and TH2) and PmTH (PmTH1 and PmTH2). Displayed are degrees of amino acid sequence identity/similarity

	HM3	HLF1V	HLF5	TH1	TH2	PmTH1	PmTH2
HV1	58/73	41/55	29/50	41/52	52/67	50/64	59/64
HM3		47/61	24/44	50/71	50/52	62/75	51/62
HLF1V			52/55	38/55	38/50	44/58	37/48
HLF5				34/43	29/38	34/46	31/40
TH1					55/64	65/78	54/65
TH2						64/70	56/70
PmTH1							57/74

**Table 2:** Lengths, numbers of acidic, basic amino acid residues, predicted isoelectric points of globular domains of hirudins (hirudin variants HV1 of Hirudo medicinalis, HM3 of Hirudinaria manillensis), hirudin-like factors (HLF1V of Hirudo medicinalis, HLF5 of Hirudinaria manillensis), tandem hirudins TH (TH1, TH2), PmTH (PmTH1, PmTH2)

Factor	Length C1–C6	Acidic/basic	pI value
HV1	34	4/2	4.41
HM3	32	5/3	4.51
HLF1	34	3/0	3.57
HLF5	25	½	6.70
TH1	32	5/5	5.59
TH2	34	5/4	4.96
PmTH1	32	5/3	4.50
PmTH2	35	4/7	8.33

Beside the central globular domain and the elongated C-terminal tail, hirudins and HLFs comprise a short N-terminal stretch of five amino acid residues immediately in front of the first cysteine residue at position 6 including a highly conserved tyrosine (or phenylalanine) residue at position 3 (see Fig. 1). The N-terminus of TH comprises either four (VCTV; assuming a signal peptide of 20 amino acid residues) or five residues (EVCTV; assuming a signal peptide of 19 amino acid residues). In both cases, the N-terminus of TH does not fit very well to the consensus sequence and lacks the conserved tyrosine or phenylalanine residue (Müller et al. 2017; Braun et al. 1988; Wallace et al. 1989; Vindigni et al. 1994; Betz et al. 1992). Strikingly, TH is the first natural representative of the hirudin family known so far that contains a tryptophan residue (see Fig. 1).

**Fig. 2:**
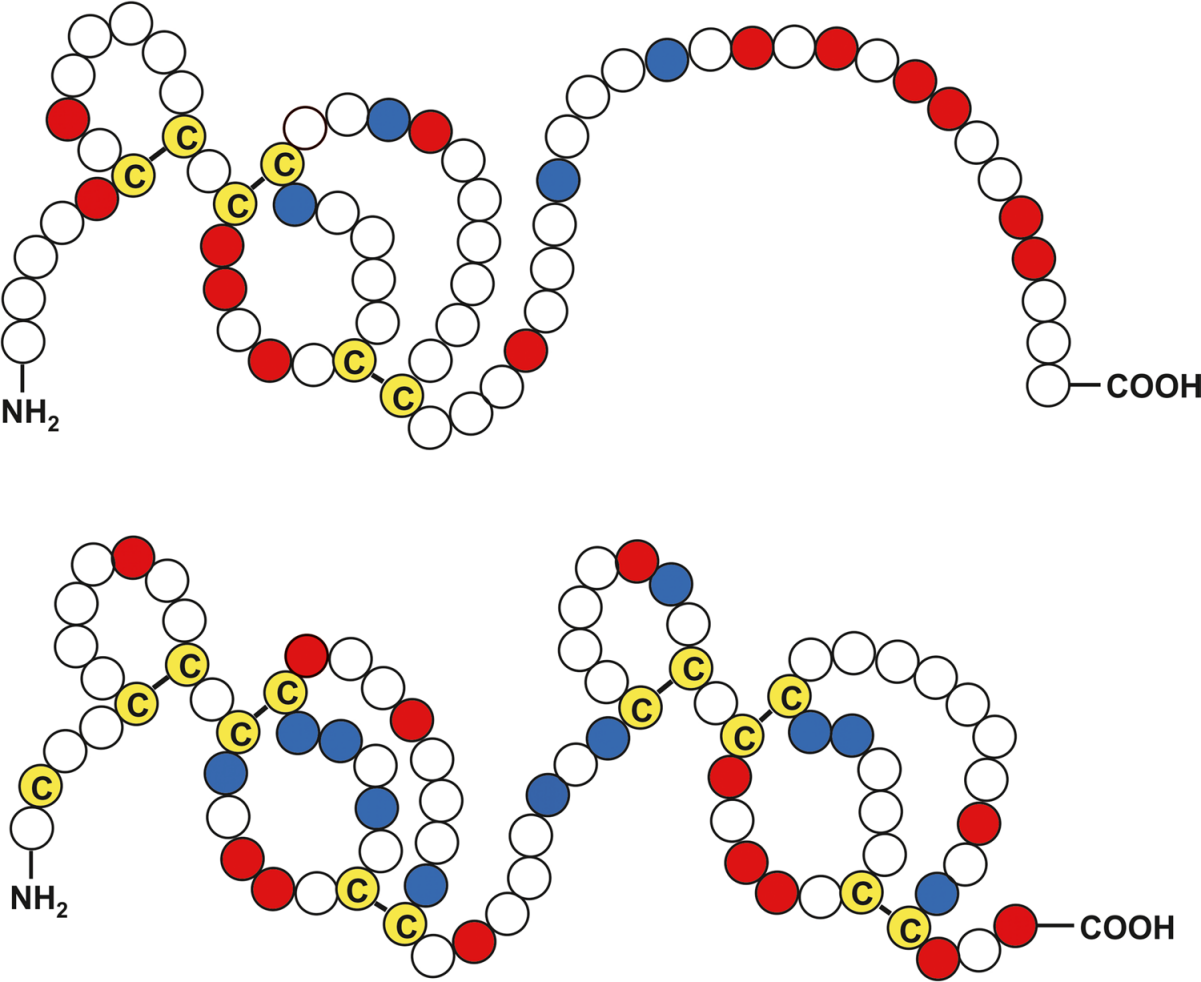
Schematic representation of HV1 (upper part) and TH (lower part). Acidic amino acid residues are labelled in red, basic amino acid residues in blue and cysteine residues in yellow. Putative disulfide bonds are indicated by bold lines

The structure of TH has not yet been determined. Structure modelling using i-TASSER (Roy et al. 2010; Yang et al. 2015) confirmed the formation of two separated globular domains, each stabilized by three disulfide bridges. According to the model, the first cysteine residue of TH at position 2 is not involved in the formation of disulfide bridges. Figure 2 illustrates the putative structure of TH in comparison to the archetype hirudin variant HV1.

### Bacterial expression and purification of TH

Bacterial expression of TH (r-TH) was performed in *E. coli* strain GM1674 according to the protocol already described earlier (Lukas et al. 2019). r-TH was expressed in large amounts; however, most of the protein could be found in inclusion bodies and only minor amounts in the cytosolic fraction. Inclusion bodies were purified and resolved in denaturing buffers containing 8 mol/l urea for further purification by His-tag affinity chromatography (Lukas et al. 2019). During dialysis at pH 8.0, the protein solution partly precipitated, and only the soluble fraction was used for further processing. However, the subsequent cleavage of the N-terminal His-tag by factor Xa digestion largely failed. Various attempts have been made to remove the remaining His-tag including the addition of up to 0.05% SDS and 0.5 mol/l urea during the cleavage reaction, increase of reaction temperature up to 37 °C and the irreversible immobilization of His-tag-TH on Co(III)-IDA (Wegner and Spatz 2013; Wegner et al. 2016), but they largely failed, too. Finally, partial sulfitolysis as described in the “Material and Method” section solved the problem. For control purposes, purification and processing by sulfitolysis was also done for hirudin-variant 1 (r-HV1).

### Functional characterisation of bacterially synthesized r-TH

**Fig. 3:**
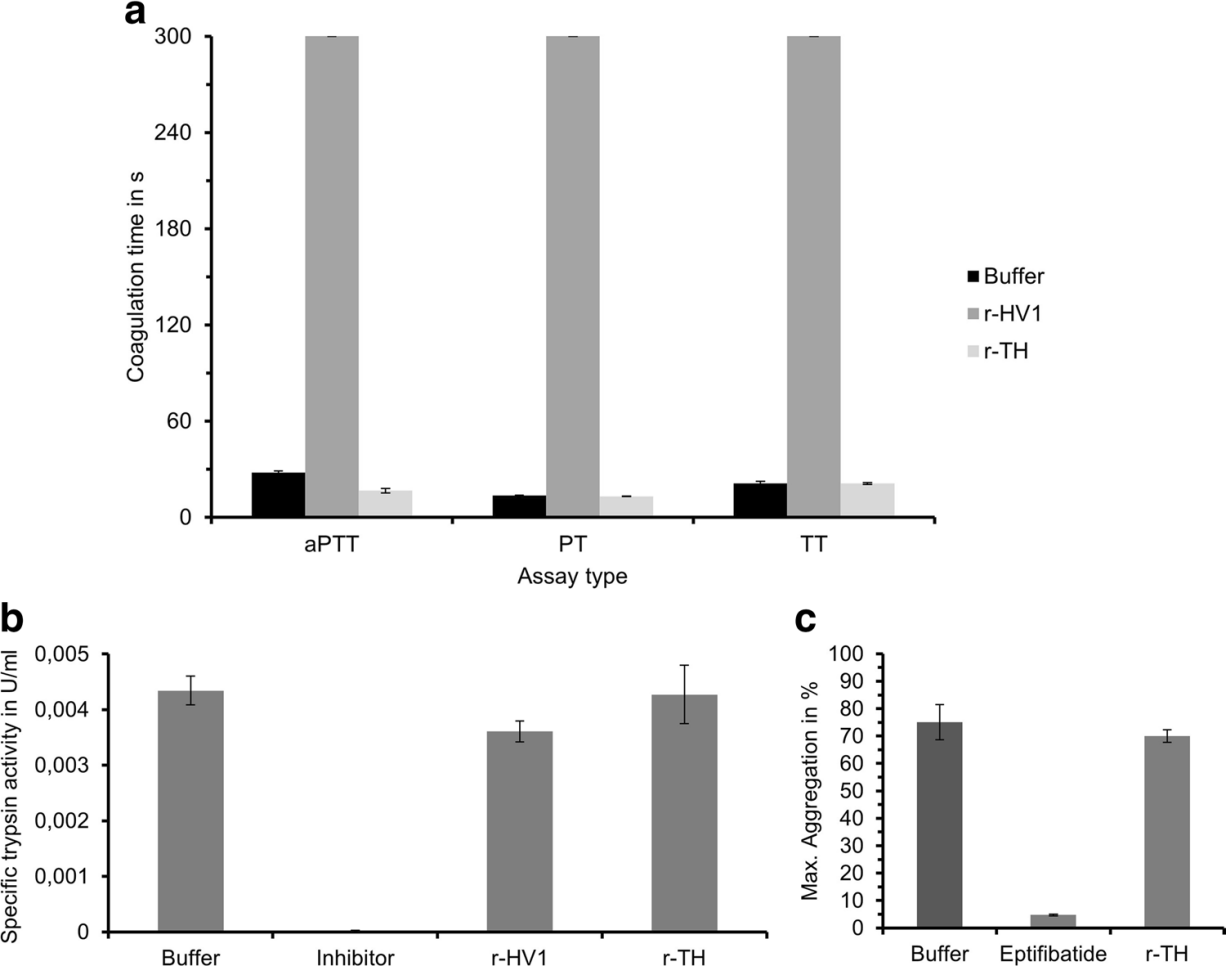
Functional characterisation of bacterially synthesized r-TH and hirudin variant 1 (r-HV1) in blood coagulation (**A**), trypsin activity (**B**) and platelet aggregation (**C**) assays. Measurements for blood coagulation were cancelled after 300 s and considered as a complete inhibition of coagulation. Concentrations used in the assays were 3.2 µmol/l (**A**), 0.4 μmol/l (**B**) and 3.8 μmol/l (**C**), respectively. aPTT indicates the activated partial thromboplastin time test, PT the prothrombin time test and TT the thrombin time test

Functional characterisation of bacterially synthesized r-TH included blood coagulation assays as well as measurements of trypsin activity and platelet aggregation. In the blood coagulation assays, r-TH was compared to equimolar amounts of r-HV1 as a positive and buffer as the negative control. No anti-coagulatory effect of r-TH could be observed in all three assays (activated partial thromboplastin time, prothrombin time and thrombin time, respectively), while r-HV1 completely inhibited blood coagulation (Fig. 3A). In addition, no inhibitory effects of r-TH and r-HV1 could be observed for either the trypsin activity or the platelet aggregation assays (Fig. 3B and C).

### Cell-free synthesis of TH in bacterial cell lysates

**Fig. 4:**
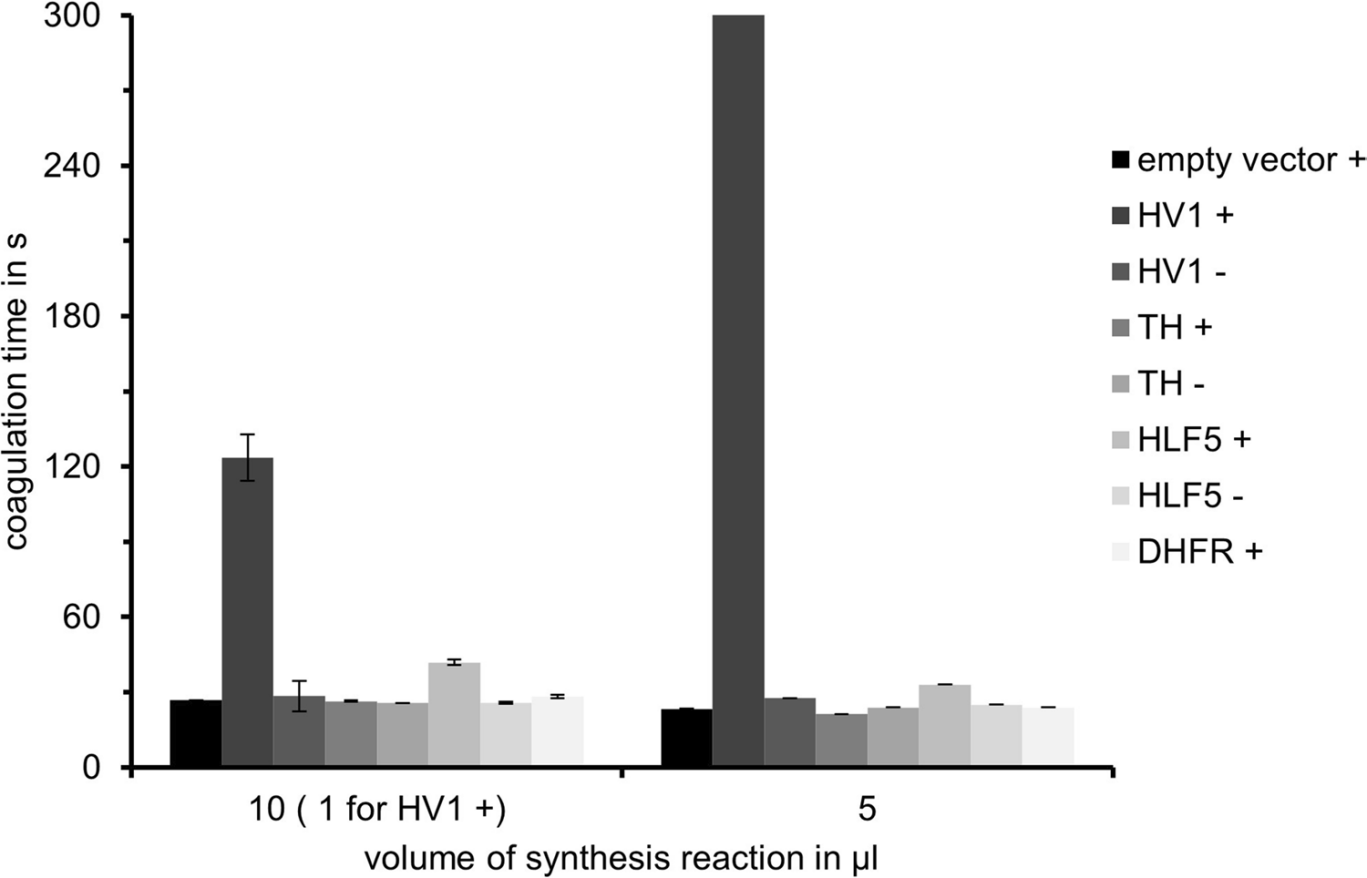
Standard blood coagulation assays using the thrombin time test (TT) of cell-free synthesized TH, hirudin HV1 and HLF5 with ( +) or without ( −) the addition of disulfide bond enhancer during the synthesis reaction in bacterial cell lysates. Lysates containing either an empty vector or a vector containing the coding region of the dihydrofolate reductase (DHFR) served as the negative controls. Aliquots of synthesis reactions used for the measurements were either 5 µl (*n* = 1, right) of 10 µl (1 µl for HV1) (*n* = 3, left)

To circumvent the procedural problems with the bacterial cell-based expression and purification of TH described above, we applied a bacteria-based cell-free protein synthesis protocol. N-terminally modified (incorporation of a methionine residue) variants of TH and hirudin variant HV1 as well as native HLF5 (a factor that naturally comprises a methionine residue at the N-terminus; see Fig. 1) were synthesized in lysates of *E. coli*. Synthesis reactions were performed with or without the addition of a disulfide bond enhancer. Aliquots of all synthesis reactions were analysed in a SDS-PAGE and tested in the thrombin time coagulation assay. As can be seen in Fig. 4, the cell lysate that contained hirudin variant HV1 revealed a very high thrombin-inhibitory potency. The potency, however, was almost entirely depended on the presence of the disulfide bond enhancer in the synthesis reaction. For HLF5, the coagulation test revealed a comparably low, but clearly detectable inhibition of thrombin. Again, the effect was dependent on the presence of the disulfide bond enhancer in the synthesis reaction. For TH, however, no inhibition could be measured, even if the disulfide bond enhancer was present in the synthesis reaction (Fig. 4).

## Discussion

More than 100 different proteins are predicted to occur in leech saliva (Baskova et al. 2004); however, most of these factors still remain uncharacterized or even unidentified. For only a few leech saliva components, functional data on one hand and molecular data on the other hand have already been assigned (Hildebrandt and Lemke 2011; Baskova and Zavalova 2001). Among them, hirudin is probably the best known and so far the only one that found its way from bench to bedside (Organisation to Assess Strategies for Ischemic Syndromes (OASIS-2) Investigators 1999; Greinacher and Lubenow 2001; Chuang et al. 2001; Frame et al. 2010; Nar 2012). Hirudin is one of the most effective natural inhibitors of thrombin, and its presence seems to be restricted to members of the order of *Hirudinida* (Müller et al. 2019a; Scacheri et al. 1993; Baskova et al. 1983; Scharf et al. 1989). Functional counterparts of hirudin in terms of thrombin inhibition, however, are present in a broad range of haematophagous animals like ticks, kissing bugs and mosquitoes as well (Koh and Kini 2010, 2009). These factors are analogous to hirudin and hence very likely the result of convergent evolution. Some of these non-leech-derived thrombin inhibitors comprise a notable structure as they are composed of two repeated domains that are arranged in a tandem-like order (Macedo-Ribeiro et al. 2008; Friedrich et al. 1993; Mans et al. 2002). Similar tandem-like structure are described for members of the leech antistasin-family including antistasin itself and ghilanten (Whitlow et al. 1999; Kim et al. 2007). Both are inhibitors of factor Xa. We describe and both functionally and molecularly characterize a novel and so far unique leech-derived factor of *Hirudinaria manillensis*, called Tandem-Hirudin (TH), that combines the core structural features of hirudin with the tandem-like arrangement found in leech antistasins and thrombin-inhibitors of ticks and kissing bugs.

### Structural properties of TH

The Tandem-Hirudin is composed of two central globular hirudin-like domains connected by a short linker sequence and contains a putative N-terminal signal peptide sequence. The presence of a signal peptide sequence was supported by all appropriate software tools; however, the exact processing side was somewhat uncertain.

The remaining N-terminal tail of TH differs both in length (four instead of five residues) and composition from respective sequences of hirudin(s) and HLFs (Müller et al. 2017). The N-terminus of hirudin blocks the reactive site of thrombin, and alterations (deletions and/or substitutions) within the N-terminus may greatly alter its thrombin-inhibitory potency (Müller et al. 2019a; Braun et al. 1988; Wallace et al. 1989; Betz et al. 1992). However, there is no strict consensus sequence of the N-terminus. But so far, all investigations concordantly confirmed that an aromatic amino acid residue at position 3 (Y3, F3 or W3) is crucial for thrombin inhibition (Lazar et al. 1991; Müller et al. 2019a). TH completely lacks a respective residue within the N-terminus. Even more strikingly, TH lacks the elongated C-terminal tail that is characteristic for all hirudins (Salzet 2000). The C-terminal tail of hirudin blocks the fibrinogen-binding site of thrombin, the so-called exosite 1 (Maraganore and Fenton 1990; Xin et al. 2015; Priestle et al. 1993). In boophilin, ornithodorin and rhodniin, the C-terminal second globular domains adopt these functions (Macedo-Ribeiro et al. 2008; Mans et al. 2002; Friedrich et al. 1993). Whether or not the same is true for TH remains to be proven. At least, the biochemical properties of TH do not seem to be prohibitive. Hirudins and HLFs with thrombin-inhibitory potencies (recently termed unorthodox hirudins) comprise an overall pI value of about 3.2–4.6, but functionality requires both an acidic central globular domain and an acidic C-terminal tail (Müller et al. 2019a; Lukas et al. 2019). The pI value of TH is about 5.03, and the respective value for the first domain is 5.59 and for the second 4.96 (Table 2). Taken together, TH displays some unique structural characteristics that are not common in hirudin(s) and HLFs and hence raised questions about its functionality.

### Genetics of TH

**Fig. 5:**
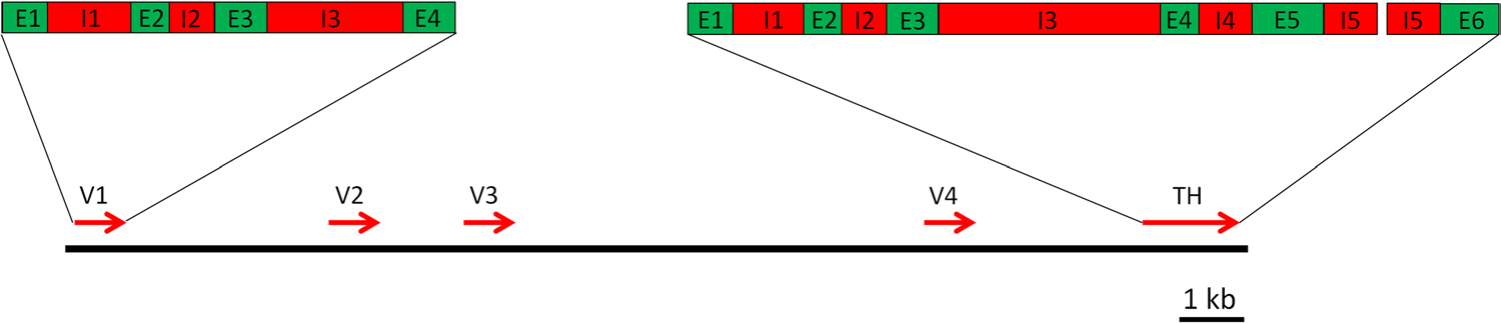
Schematic representation of a segment on genome contig00065 of *Hirudinaria (Poecilobdella) manillensis* comprising four putative hirudin/HLF genes (Hiru_V1–V4) and one putative tandem hirudin gene (PmTH). Red arrows indicate position, size and orientation of the respective genes. The upper boxes illustrate the structures of two genes (left: putative hirudin/HLF Hiru_V1; right: putative tandem hirudin PmTH). Exons (E) are labelled in green, and introns (I) are labelled in red. The bar indicates a length of 1 kb

The genes of hirudins and HLFs comprise a common structure of four exons and three introns (Scacheri et al. 1993; Müller et al. 2017, 2019a). The first exon encodes the signal peptide, the second and the third exon encode the short N-terminal stretch and the central globular domain, whereas the fourth exon encodes the elongated C-terminal tail. Despite intensive efforts, we failed to identify and characterize the gene encoding TH in our specimen of *Hirudinaria manillensis*. However, when thoroughly analysing the genome data of *Hirudinaria* (*Poecilobdella) manillensis* (Guan et al. 2020; GenBank Acc. No. SRX4286737), we identified a segment of about 18,200 bp in length on contig00065 (GenBank Acc. No. JADEYA010000065, position 1,28,3800 to 1,302,000) that contained the genes of four putative hirudins/HLFs and the gene of a putative TH (PmTH, see also Fig. 1). The respective segment was annotated and deposited as a Third Party Annotation (TPA) in GenBank under Acc. No. BK059527. Figure 5 summarizes the genetic structure of the respective segment in the *Hirudinaria* (*Poecilobdella*) *manillensis* genome. The putative PmTH gene comprises six exons and five introns, whereby the exons 4 and 5 exhibit the same structure as the exons 2 and 3 and are hence most likely the result of a duplication event.

**Fig. 6:**
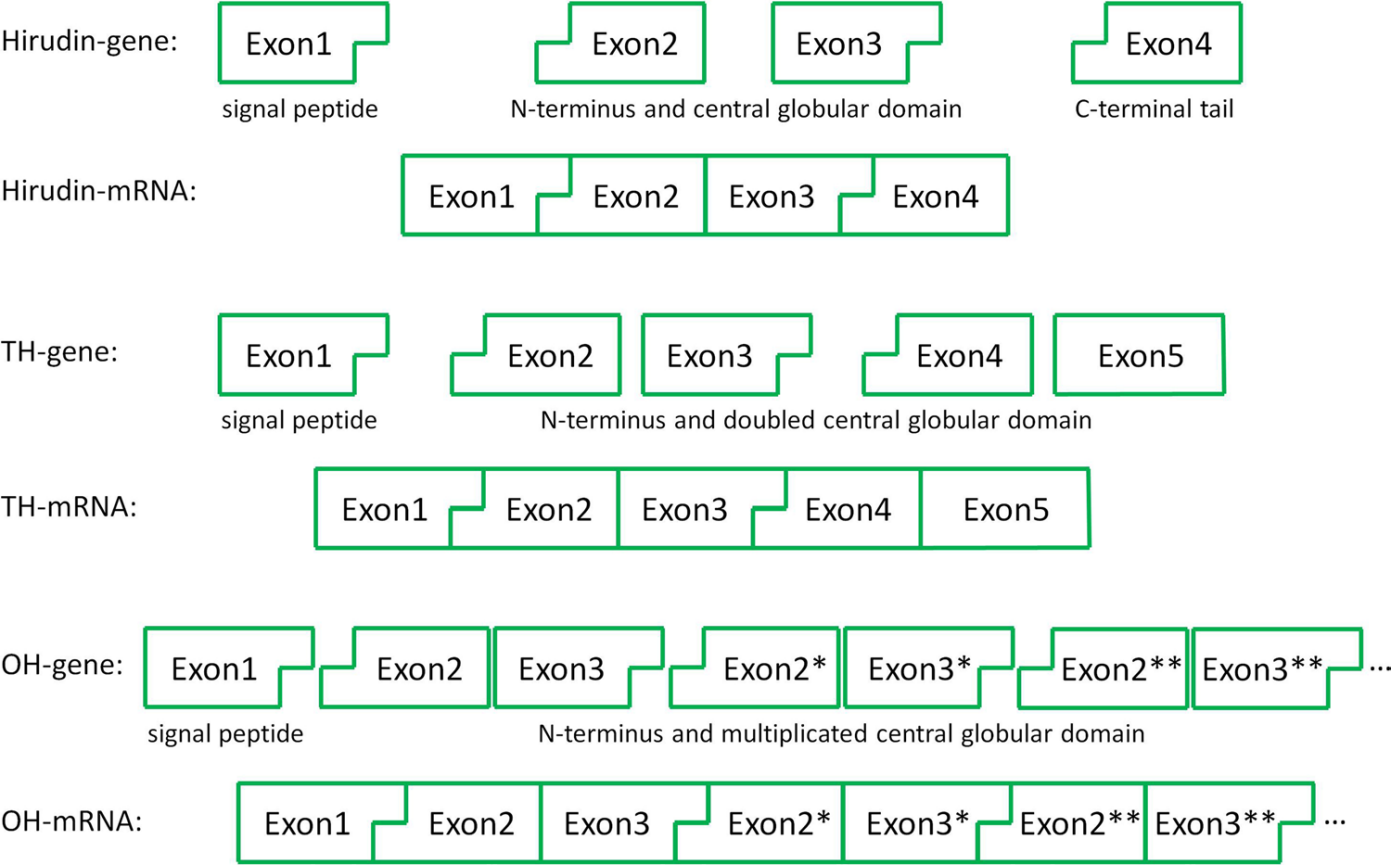
Schematic drawing visualizing the multiplication hypothesis. TH indicates the tandem hirudin, OH a putative molecule with multiple hirudin-like domains. Exon-overlapping codons are illustrated by an angled connection

The exact composition of exons 2 and 3 supports such a duplication (or even multiplication) scenario. Exon2 of hirudin and the HLF genes starts with the second nucleotide of a triplet that codes for the first amino acid residue of the mature factors (the first nucleotide of the respective triplet is provided by exon 1). Exon 3 ends with the first nucleotide of a triplet that is completed by the first two nucleotides of exon 4 (in hirudin and HLF genes) or by the duplicated exon 2 (= exon 4) in PmTH. Further rounds of such exon duplication events would lead to the formation of molecules with multiple hirudin-like domains. A scheme visualizing the multiplication hypothesis is shown in Fig. 6.

Comparable scenarios of multiplication have been described for members of the antistasin-family in *Hydra* sp. (Holstein et al. 1992) or in the disc abalone *Haliotis discus discus* (Nikapitiya et al. 2010).

An alternative way to generate a TH-encoding mRNA would be trans-splicing. RNA trans-splicing combines two separate pre-mRNA molecules to form a chimeric non-co-linear RNA molecule (Lei et al. 2016). So far, trans-splicing has been described in several groups of eukaryotes including trypanosomes, nematodes, insects and even mammals, but no yet in leeches. However, the exploitation of such a mechanism would have the potential to greatly enhance the variability of hirudins and HLFs in these animals.

The presence of TH genes outside the genus *Hirudinaria* (*Poecilobdella*) remains to be proven. In our own analysis, we did not find any evidence for TH genes in the genomes of leech species like *Hirudo medicinalis* (Kvist et al. 2020; Babenko et al. 2020), *Whitmania pigra* (Tong et al. 2022) or *Helobdella robusta* (Simakov et al. 2013). The identification of additional TH and/or OH genes is a prerequisite for any further investigations on the origin of TH genes in an evolutionary context.

### Synthesis and functional characterization of TH

We have multiple times successfully used bacterial expression systems for the large-scale synthesis of His-tagged hirudin(s) and HLFs that were subsequently purified by affinity chromatography and functionally validated (Müller et al. 2016, 2019a, 2019b; Lukas et al. 2019). For TH, this approach failed. TH was very well over-expressed by the bacteria, but was almost completely found in insoluble inclusion bodies. Intense efforts have been made to reconstitute functionally active TH. Whereas the His-tag of TH could be successfully removed, the final TH did not show anti-coagulatory potency or any other inhibitory effects (Fig. 3). Two possible scenarios could explain these results. First, TH does not comprise these activities at all. Second, TH may not be in its correct and hence functional conformation (Chang et al. 1995). As described above, TH comprises 13 cysteine residues. Six pairs of these residues are supposed to be involved in the formation of disulfide bridges, three within the first and three within the second repeat of the tandem globular domains (see Fig. 2). The bacterial expression and in vitro processing of TH by sulfitolysis and refolding may likely have not resulted in the correct formation of all these several disulfide bridges (Thomas and Baneyx 1996; Baneyx and Mujacic 2004; García-Fruitós et al. 2016; Mukherjee and Gupta 2016). Mathematical calculations reveal a number of 135,135 different combinations of disulfide bridges based on 13 cysteine residues (and one remaining free SH group) (Combet et al. 2000). Clearly, not all of the calculated combinations are sterically possible, but nevertheless, the number indicates the very low probability of a correct folding simply by chance (Zhang and Snyder 1989). To improve the probability of correct folding, we decided to use cell-free protein synthesis approaches.

To our knowledge, the bacterial cell lysate-based approach has never been before applied for the synthesis of hirudins in particular or leech saliva components in general. Our data clearly indicate that both hirudin variant HV1 of *Hirudo medicinalis* and HLF5 of *Hirudinaria manillensis* were synthesized in biologically active forms (see Fig. 4). The observed thrombin-inhibitory potency, however, was completely dependent on the presence of a disulfide bond enhancer in the synthesis reaction, once again highlighting the crucial importance of correct disulfide bond formation and folding of hirudins and HLFs for their biological activities. For TH, no inhibitory effect on thrombin activity could be observed, even when the disulfide bond enhancer had been present in the synthesis reaction mixture.

Further large-scale synthesis and purification approaches will hopefully result in higher yields of TH to perform additional functional assays to confirm the present results and/or identify the actual biological target of TH. In addition, it is without any doubt of great interest to determine the structure of TH in a complex with its biological target.

## Summary

The current study describes the identification and partially successful synthesis of a putative secretory protein of *Hirudinaria manillensis*. The protein comprises a structure that has not yet been described for members of the hirudin superfamily: two central globular domains of hirudin are arranged in a tandem-like way. The protein was hence termed Tandem-Hirudin (TH). Several functional tests including coagulation assays, platelet aggregation assay and a trypsin activity assay did not yet reveal the biological target of TH.

## Data Availability

All relevant sequence data were deposited in GenBank for public access.
